# The covert symphony: cellular and molecular accomplices in breast cancer metastasis

**DOI:** 10.3389/fcell.2023.1221784

**Published:** 2023-06-27

**Authors:** Hongjiang Si, Madelyn Esquivel, Erika Mendoza Mendoza, Kevin Roarty

**Affiliations:** ^1^ Department of Molecular and Cellular Biology, Baylor College of Medicine, Houston, TX, United States; ^2^ Dan L. Duncan Comprehensive Cancer Center, Baylor College of Medicine, One Baylor Plaza, Houston, TX, United States

**Keywords:** breast cancer, tumor microenvironment, CTC (circulating tumor cells), dormancy, metastasis (cancer metastasis), metastatic niche

## Abstract

Breast cancer has emerged as the most commonly diagnosed cancer and primary cause of cancer-related deaths among women worldwide. Although significant progress has been made in targeting the primary tumor, the effectiveness of systemic treatments to prevent metastasis remains limited. Metastatic disease continues to be the predominant factor leading to fatality in the majority of breast cancer patients. The existence of a prolonged latency period between initial treatment and eventual recurrence in certain patients indicates that tumors can both adapt to and interact with the systemic environment of the host, facilitating and sustaining the progression of the disease. In order to identify potential therapeutic interventions for metastasis, it will be crucial to gain a comprehensive framework surrounding the mechanisms driving the growth, survival, and spread of tumor cells, as well as their interaction with supporting cells of the microenvironment. This review aims to consolidate recent discoveries concerning critical aspects of breast cancer metastasis, encompassing the intricate network of cells, molecules, and physical factors that contribute to metastasis, as well as the molecular mechanisms governing cancer dormancy.

## Introduction

### Breast cancer: a snapshot

Approximately 341,960 new cases of breast cancer were estimated in the United States in 2022 (Giaquinto et al., 2022), and about one in eight women are diagnosed with breast cancer in their lifetime. Roughly 65% of patients develop hormone receptor–positive (HR+) breast cancer, characterized by the presence of estrogen receptor (ER) and/or progesterone receptor (PR). Meanwhile, 20% of patients exhibit amplification of the human epidermal growth factor receptor 2 (HER2+, HR-). The remaining 15% of breast cancer patients that do not express these receptors are diagnosed with triple-negative breast cancer (TNBC; ER-, PR-, HER2-) (Loibl et al., 2021). The evolution of breast cancer classification, which was once solely reliant on histology, now encompasses a more comprehensive approach by integrating clinical symptoms and tissue-specific biomarkers, marking a significant advancement in understanding and diagnosis of the disease (Loibl et al., 2021). The seminal work of Perou and Sorlie in 2000 introduced an intrinsic classification that identified four distinct breast cancer subtypes: luminal A and luminal B (both characterized by the presence of the ER), along with the basal-like and HER2-enriched subtypes (devoid of ER expression). This innovative classification system sparked a paradigm shift in the clinical management of breast cancer, moving from a focus on tumor size to an approach rooted in biological features (Perou et al., 2000; Sorlie et al., 2001). Typically, the largest cluster of breast cancers consist of ER-positive (ER+) tumors, divided into luminal A and B subgroups, with luminal B showing higher cell proliferation gene expression. Another cluster comprises mostly ER-negative (ER-) tumors, further categorized as TNBC or basal-like tumors, and the HER2-enriched group. The TNBC subtype has undergone further refinement, resulting in the identification of distinct subclasses: mesenchymal (M), mesenchymal stem-like (MSL), luminal androgen receptor (LAR), and the basal-like BL1 and BL2 subtypes (Lehmann et al., 2016).

Collectively, this molecular stratification into subtypes now helps inform treatment strategies, and in a large fraction of patients with early-stage disease, present-day treatments can be curative in 70%–80% of patients (Loibl et al., 2021). Management of early-stage breast cancer fundamentally relies on an evaluation of the tumor load and its specific subtype (Waks and Winer, 2019; Loibl et al., 2021). Surgery is generally succeeded by adjuvant hormone therapy in all instances of estrogen receptor-positive (ER+) diagnosis. When patients present a high risk of relapse, as determined by gene expression signature or node status, chemotherapy might be advised. Neoadjuvant subtype-specific systemic therapy, followed by surgery, is the conventional treatment protocol for cases of TNBC and HER2+ early breast cancer. For HER2-positive early breast cancer, including both luminal-like and non-luminal-like variations, the typical treatment method now involves neoadjuvant chemotherapy coupled with anti-HER2 treatment. For TNBC, the generally accepted treatment method includes chemotherapy, usually with the inclusion of an anthracycline and a taxane. Nevertheless, for TNBC patients with a limited disease load, docetaxel and cyclophosphamide have proven to be equally efficacious and may be considered as alternative options if the use of anthracyclines needs to be avoided.

Unlike early-stage breast cancer, advanced breast cancer, though treatable, is considered incurable and is responsible for the vast majority of cancer-related deaths (Chaffer and Weinberg, 2011). Among HR+/HER2- patients, the 5-year survival rate drops from 90.1% for those with regional disease to 31.9% for those with distant metastases. For HER2+ patients the difference between regional and distant disease is either 89.3%–46% (HR+) or 82.8%–38.8% (HR-). For TNBC, which disproportionally affects women under 40, African American, and Hispanic patients (Dietze et al., 2015; Rey-Vargas et al., 2019), this 5-year survival rate drops from 65.8% to 12% (SEER, 5-year relative survival percentage, 2012–2018). Of the molecular subtypes of breast cancer, TNBC displays a higher degree of heterogeneity at the genomic, transcriptomic, and mutational level (Kudelova et al., 2022). Access to large patient databases such as The Cancer Genome Atlas (TCGA) and the Molecular Taxonomy of Breast Cancer International Consortium (METABRIC) has promoted further research into gene-ontology analysis and subtyping analysis methods to identify targetable driver mutations of treatment resistance and recurrence in TNBC patients (Asleh et al., 2022). Importantly, the underlying classification system of breast cancers, which is fundamentally based on their specific biological characteristics, plays a significant role in shaping the profile of metastatic disease. This includes aspects such as the timing at which metastasis occurs and the specific body sites to which the cancer cells spread. Therefore, understanding the processes that govern metastatic progression is essential for improving survival outcomes in patients.

### Breast cancer metastasis at a glance

**FIGURE 1:**
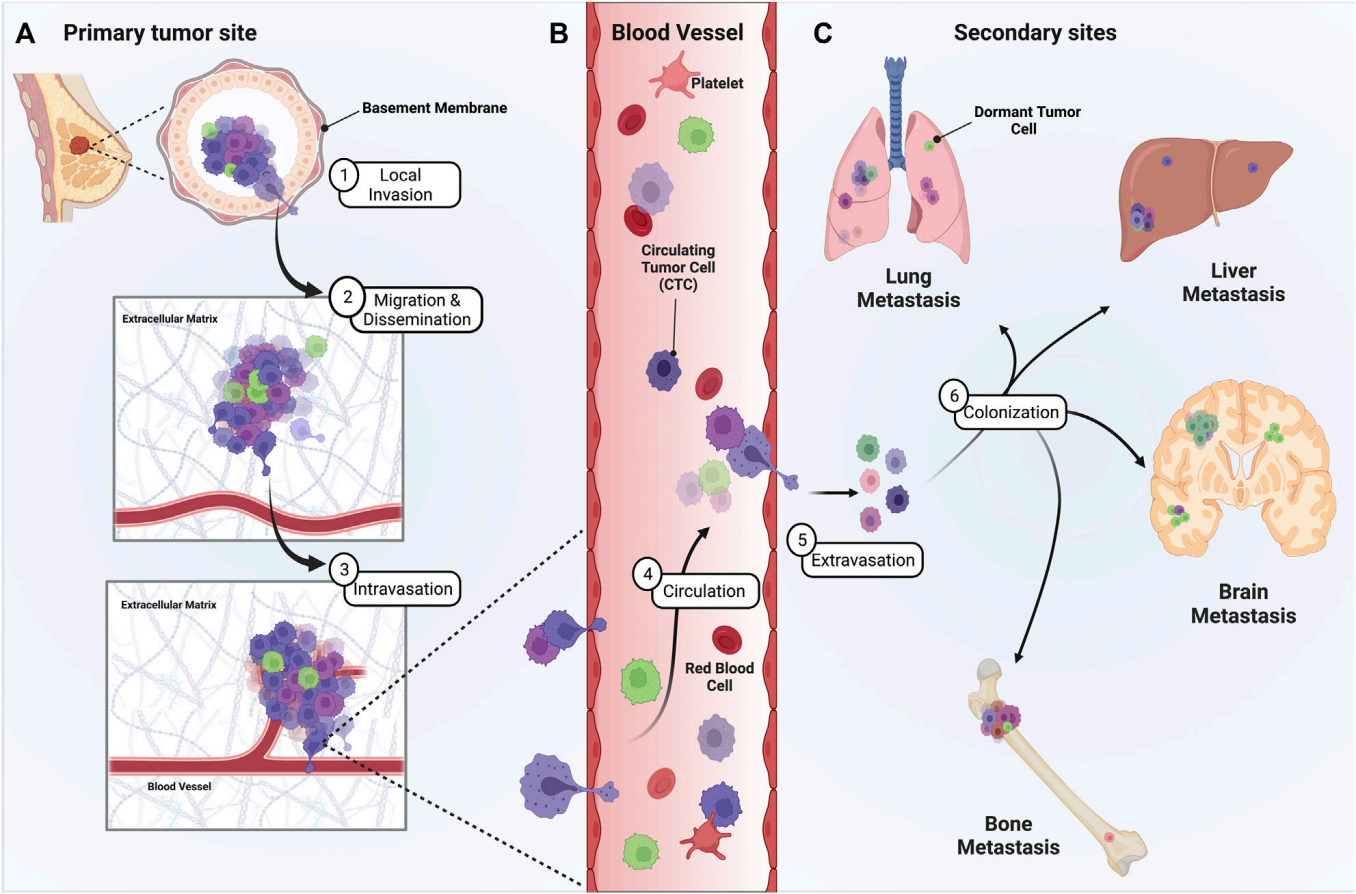
Metastatic progression of breast cancer from the primary tumor site to distant secondary sites is a multistage process. Presented herein is a graphic depicting the steps of breast cancer metastasis. **(A)** To metastasize, heterogenous tumor cells must leave the primary tumor site, often by invading through the ductal basement membrane, migrating through the ECM, and intravasating into circulation. **(B)** Once in circulation, single CTCs and/or CTC clusters must survive the shear stress and immune microenvironment of the vasculature until they adhere along the vessel wall at distant sites. **(C)** Eventually, disseminated tumor cells extravasate, proliferate, and colonize distant sites of the lung, liver, brain, and/or bone. However, some tumor cells can remain dormant at these secondary sites, representing a significant risk and clinical challenge for breast cancer patients.

In metastasis, cells initially disseminate from the primary tumor and locally invade the surrounding tissue (Figure 1A). Disseminated cells that intravasate into the circulatory and/or lymphatic vessels, known as circulating tumor cells (CTCs), can then travel to distant organs (Figures 1B,C). Beyond survival in the circulation, the final destination of disseminated tumor cells relies on their ability to adhere to the microvasculature wall at a distant site, extravasate/exit the vessel, and finally colonize secondary organs (Chaffer and Weinberg, 2011) (Figures 1B,C). The central theory surrounding metastatic progression remains Stephen Paget’s “seed and soil” hypothesis (Paget, 1989). Paget theorized that cancer cells (seeds) intravasate and circulate to distant organs (soil) and form secondary metastases in environments capable of supporting colonization, with the idea that certain “soils” supported specific “seeds” better than others. Isaiah Fidler experimentally validated this theory in 1976 using melanoma cell variants to show selectivity of organ seeding (Fidler and Nicolson, 1976). This preferential seeding in certain organs over others, known as organ tropism, is observed in the setting of breast cancer. Clinical proof for the existence of organ tropism is best demonstrated by the observation that intrinsic molecular classification of breast cancers, originally defined by Perou and Sorlie (Perou et al., 2000), can dictate both the timing and location of metastatic disease. For instance, the timing of metastatic relapse differs by subtype, with luminal A relapses typically occurring between 5–15 years after initial presentation and TNBC tumors relapsing within the first 5 years (Kennecke et al., 2010). Most breast cancer subtypes can metastasize to bone, yet HR+/HER2+ luminal tumors have a higher propensity to metastasize to bone over other subtypes (Chu and Allan, 2012). Her2-enriched (HR−/HER2+) tumors exhibit a higher probability to metastasize to brain and liver, while TNBC tumors preferentially hone to the lungs (Wu et al., 2017). Of course, the concept of the “seed and soil” hypothesis has continued to evolve. Researchers have come to appreciate that metastatic progression is driven by a combination of intrinsic and extrinsic factors that can condition both tumor cells and their surrounding microenvironment to establish an ecosystem known as the metastatic niche. Both tumor cell intrinsic and extrinsic microenvironmental factors contribute to the initial dissemination and eventual establishment of distant metastasis. Cell intrinsic factors inherent to tumor cells during metastasis include, but are not limited to, 1) cellular composition of a tumor, referred to as intratumoral heterogeneity, 2) plasticity between phenotypic cellular states, 3) resistance to anoikis and apoptosis, and 4) metabolic adaptation.

The development of newer technologies like single cell RNA sequencing (scRNA-seq) are providing an unprecedented level of molecular refinement on top of previous work to capture both spatial and molecular information across the breast cancer landscape (Wu et al., 2021). Despite the profound impact of molecular diversity driving present-day treatments, advanced breast cancers remain incurable, and the application of such technologies like scRNA-Seq in the context of metastatic disease are still evolving. In this review, we provide an overview of some key cellular processes contributing to metastasis, molecular players that make up the metastatic niche, and important signaling networks that mediate both dormancy and emergence of breast cancer metastases at distant sites.

Beyond the intrinsic molecular heterogeneity seen in breast cancers, precisely how tumor cells escape and survive the unchartered landscape of metastasis remains unclear. Acclimatization or adaptation by tumor cells is a prerequisite for successful metastasis. Tumor cells must exhibit a level of cellular plasticity, enabling them to change their cellular state and functional behaviors to disseminate, survive, and colonize a distant organ. Often these cell-state transitions are bidirectional and can be transient or permanent depending on the context and degree of engagement. The epithelial-to-mesenchymal (EMT) transition instructs epithelial cells during embryonic development, tumor progression, or wound healing to acquire more mesenchymal traits (Nieto et al., 2016). This process was originally appreciated in vertebrate embryogenesis (Hay et al., 1968) and later recognized in the context of cancer for tumor cells to invade and metastasize. In this process, cells lose key cell-cell adhesion proteins, such as the adherens junction, E-cadherin (Cdh1) and gain a spectrum of mesenchymal markers (N-cadherin, Vimentin) through the activation of a core transcription factor (EMT-TF) repertoire (Snail, Slug, TWIST1, ZEB1/2) (Basu et al., 2018). The outcome of EMT is the acquisition of a mesenchymal state; however, intermediate states exist within this transition that engender tumor cells with a spectrum of partially activated mesenchymal properties which support invasion and dissemination, albeit to different extents (Heerboth et al., 2015; Pastushenko and Blanpain, 2019). This diversity in the spectrum of cell states thus creates a level of phenotypic complexity that warrants further investigation, especially in the context of metastasis. Multiple extracellular signals like Transforming Growth Factor-β (TGF-β), Wnt, and Hepatocyte Growth Factor (HGF) pathways initiate a mesenchymal program under different contexts by engaging core EMT-TFs. Exposure to such EMT or MET modulators in metastasis could greatly shape tumor cell fitness and survival. The signaling cues and networks that drive such cell state changes remain a significant area of investigation.

CTCs comprise a subgroup of tumor cells that detach from the primary tumor and enter the bloodstream. Interestingly, very few CTCs can complete the requisite steps of metastasis, yet there is a need to understand the biology of such disseminated cells given that a subset represents the precursor to metastatic disease. CTC detection in the circulation is linked to poor clinical outcome, and such cells have been detected as single cells or multicellular aggregates comprised of either homotypic or heterotypic partners (Cristofanilli et al., 2004; Aceto et al., 2014; Wang et al., 2017; Lo et al., 2020). In breast cancer, the number of CTCs detected in the circulation can be prognostic (Paoletti et al., 2019). Though CTCs are typically detected as single cells in the circulation, clustered forms typically metastasize better in experimental settings, though less readily apparent in the circulation (Aceto et al., 2014). These multicellular CTCs, previously assumed to be too large for capillary transit, can reorganize into chain-like structures to navigate narrow vessels successfully, suggesting a more significant role for CTC clusters in tumor dissemination and potential therapeutic strategies targeting these clusters to combat metastasis (Au et al., 2016). Moreover, cell state composition of CTCs, based on epithelial and mesenchymal characteristics, exists across intrinsic breast cancer subtypes. In a study of 41 patients, 17 tested positive for CTCs, and the manifestation of EMT traits in these CTCs varied based on the histological subtype of the cancer (Yu et al., 2013). Most of the CTCs in patients with lobular type (typically ER+/PR+) cancers were epithelial, while in patients with TNBC, the CTCs were largely mesenchymal. Surprisingly, HER2+ breast cancer patients showed a dominance of mesenchymal CTCs. Interestingly, CTC clustering was shown to regulate the DNA methylome by impinging on master transcriptional regulators of stemness like OCT4, NANOG, SOX2 and SIN3A (Gkountela et al., 2019). In other studies, epithelial features were associated with a positive chemotherapy response after longitudinal monitoring of EMT features in CTCs, while mesenchymal features were typically detected in CTCs upon therapeutic relapse (Yu et al., 2013; Kvokačková et al., 2021). These observations are in line with emergent mesenchymal traits exhibited in primary residual disease settings following neoadjuvant chemotherapy (Creighton et al., 2009). Importantly, the emergence of CTC clusters in breast cancer metastases can also occur through a process involving the local spread of a multicellular cluster of K14+ cancer cells from the primary tumor, their subsequent entry into the bloodstream, and eventual establishment in distant locations (Cheung et al., 2016). CTCs also coexist with other cell types in the circulation, underscoring the interactive biology within this step of metastasis (Duda et al., 2010; Szczerba et al., 2019). The association of breast cancer CTCs with other cells like hematopoietic cells (Aceto et al., 2014; Sharma et al., 2021), fibroblasts (Matsumura et al., 2019; Ortiz-Otero et al., 2020; Sharma et al., 2021), additional tumor cells, or endothelial cells (Taftaf et al., 2021) imparts several advantages for CTCs in the circulation, including 1) the availability of growth factors for cell survival, proliferation, and immune evasion and 2) the establishment of intercellular adhesions to prevent anoikis and cell death.

Metastasis critically hinges upon the intricate coordination between tumor cells and their microenvironment at each stage. Though significant strides have been made in identifying pivotal interactions that influence metastatic activities of tumor cells, our comprehension of metastasis remains somewhat incomplete. A wealth of research has probed into the distinct physiological traits of the local cellular microenvironment, also known as the ‘niche’, of primary tumor cells, and the cells in metastatic sites. The subsequent segments offer a comprehensive analysis of the essential cellular and molecular elements thought to engage and create the conducive environment that defines the metastatic microenvironment.

## The tumor microenvironment (TME) in metastasis

**FIGURE 2:**
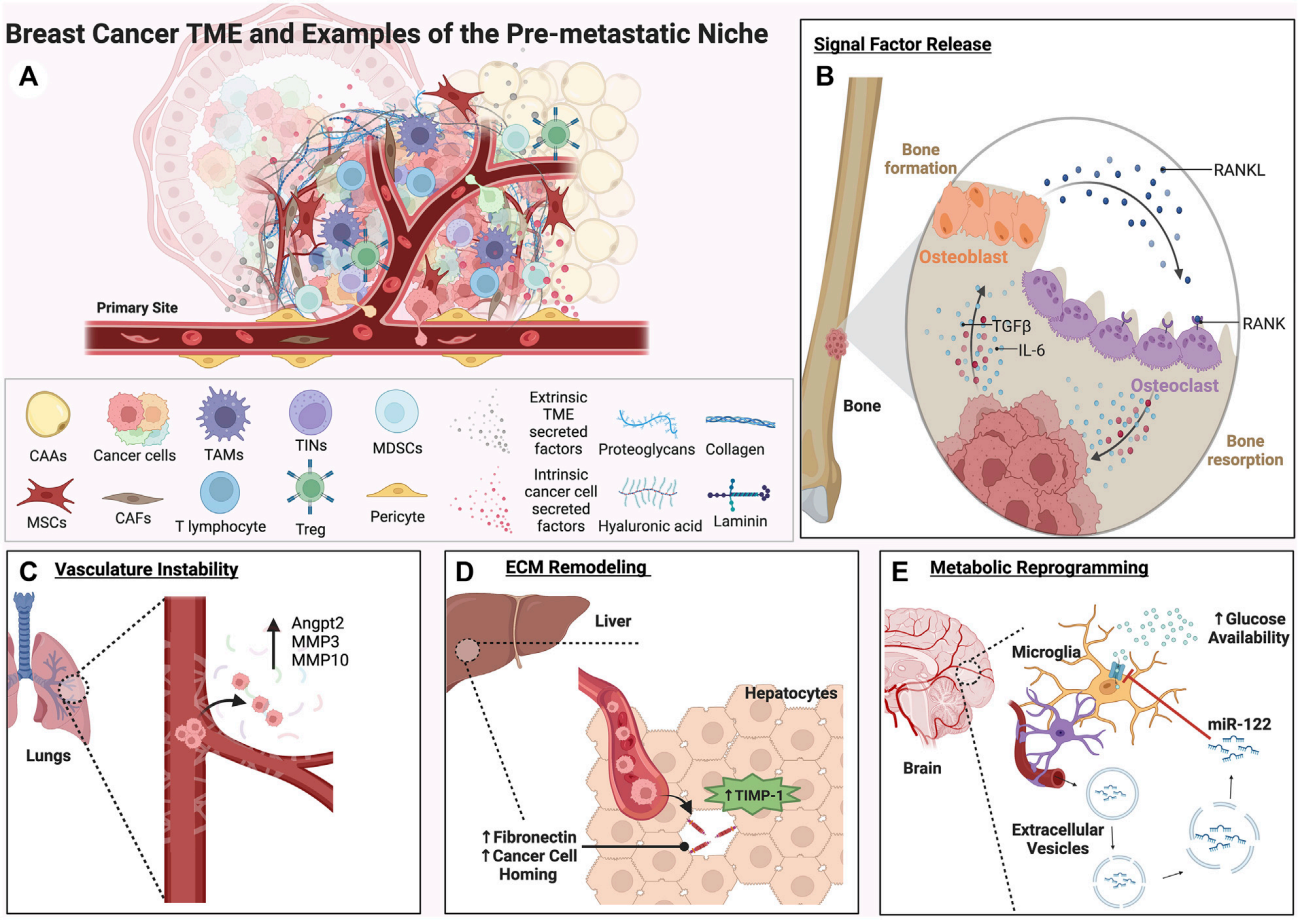
A complex roster of both tumor cell intrinsic and extrinsic cues within the TME can facilitate the formation of the pre-metastatic niche. Factors released from both tumor cells and the TME can activate signaling pathways involved in plasticity, proliferation, and migration within the primary tumor and distant secondary sites of metastasis. These examples represent some of the many ways secondary sites can be conditioned to form an ideal metastatic niche for disseminated tumor cells. **(A)** A graphical depiction of the primary tumor site showing the potential complexity of the breast cancer TME and key cell types that contribute to metastatic progression. **(B)** The bone metastatic niche is shown. The RANK/RANKL vicious cycle of bone formation and resorption is depicted in **(B)**. Through this process, growth factors such as TGFβ and inflammatory cytokines like IL-6 are released, driving EMT and facilitating pre-metastatic niche formation in the bone. **(C)** Matrix metalloproteinases and vasculature remodeling factors expressed by tumor cells can contribute to vasculature instability and extravasation in the lungs. (D) In the liver, TIMP-1 secreted by hepatocytes can trigger remodeling of the ECM and homing of tumor cells to a fibronectin and TGFβ rich microenvironment (E). Extracellular vesicles containing the miRNA miR-122 secreted by tumor cells can travel through the vasculature to the brain, downregulating glucose uptake and creating a nutrient rich environment for metastases.

Cooperation between tumor cells and non-malignant cells in the TME represents a major area of study regarding metastatic progression of solid tumors like breast cancer. The TME consists of a diverse repertoire of stromal cell types and non-cellular extracellular matrix (ECM) components that provide a rich cellular and structural landscape for tumor cells (Figure 2A). Collectively, such components lend a supportive function for tumor cells by supplying growth factors, chemokines, and other secreted factors that shape both the primary and distant metastatic niche (Hill et al., 2020). Importantly, while the tumor cell itself is central to the formation of distant metastases, the cellular topography encountered by tumor cells, and the resultant heterotypic cell-cell interactions occurring within the TME, vastly changes throughout the course of disease progression. Some key stromal cell types that contribute to metastasis and coordinate reciprocal interactions with tumors cells include tumor-associated mesenchymal stem cells, cancer-associated fibroblasts (CAFs) neutrophils, macrophages, adipocytes, endothelial cells, pericytes, and other organ-specific players that interact with tumor cells through complex signaling networks (Eiro et al., 2019; Hill et al., 2020). In the following section, we highlight some of these key cell types and their role within the TME during breast cancer metastasis.

### Mesenchymal stem cells

Mesenchymal stem cells (MSCs) are a type of multipotent stem cell originally found in the bone marrow but also discovered in other locations like fat tissue, cord blood, and more. They play key roles in the upkeep and restoration of an array of connective tissues such as bone, adipose tissue, cartilage, and muscle (Nombela-Arrieta et al., 2011). While the bone marrow is the primary reservoir for MSCs, they can also be found in numerous other tissues. Importantly, MSCs have long been implicated in the maintenance of pro-metastatic states in breast cancer (Hill et al., 2017). The comparison of cancer to “a wound that never heals” is a concept born of MSCs and their capacity for self-renewal (Pittenger et al., 1999). Like a wound, MSCs are recruited to the TME by signaling factors secreted by tumor cells. Karnoub *et al.* originally demonstrated that MSCs derived from bone-marrow elicit a reciprocal CCL5/CCR5 paracrine loop between MSCs and tumor cells to enable metastatic competence of otherwise indolent tumor cells (Karnoub et al., 2007). Chaturvedi *et al.* further implicated hypoxia-induced factors (HIFs) as a trigger for MSC homing to primary tumors, facilitated in part by CCL5/CCR5 and an additional reciprocal MSC-tumor cell feedback loop promoting placental growth factor (PGF) expression by breast tumor cells to reinforce MSC recruitment, angiogenesis, and increased lung and lymph node metastasis (Chaturvedi et al., 2012). MSCs are also known to promote invasion and self-renewal properties of tumor cells, and inhibition of MSC homing can reduce the metastatic potential of breast cancer cells (Camorani et al., 2017). More recently, these reciprocal tumor-MSC interactions have also been implicated in chemoresistance in breast cancer through “secretome-educated” heterogeneity, emphasizing the clinical importance of deconstructing the signaling pathways that drive such interactions (Roodhart et al., 2011; Plava et al., 2019).

### Cancer associated fibroblasts

In the context of development, fibroblasts predominantly generate connective tissue ECM, and studies indicate that this function evolves as aging or disease-related states ensue (Plikus et al., 2021). Fibroblasts are also integral to tissue repair, becoming more active in response to tissue injuries. In the context of cancer, a definitive molecular characterization for CAFs does not exist; however, such cells exhibit an extended shape and lack markers typical of epithelial, endothelial, and leukocyte cells, all while being void of mutations characteristic of cancer cells (Kalluri, 2016). It is conceivable that essential characteristics of CAFs mirror the conventional physiological functions undertaken by fibroblasts. CAFs are often characterized by their expression of smooth muscle actin (SMA⍺), fibroblast specific protein (FSP1), platelet derived growth factor receptor β (PDGFRβ), and are known for their role in remodeling the ECM (Yamashita et al., 2012; Chen and Song, 2019). Human tissue-based studies have highlighted the stepwise changes within the fibroblastic stroma, which is often characterized by early fibroblast expansion prior to malignancy, and their common presence around preliminary or pre-cancerous lesions. This slow metamorphosis implies that the bulk of CAFs are derived from local fibroblasts under tissue distress. Experimental evidence also suggests that other cell types can give rise to CAFs in some contexts. For instance, MSCs from bone marrow can transdifferentiate into CAFs *in vivo* after their recruitment to primary tumors and lung metastases (Weber et al., 2015; Raz et al., 2018). Contrary to resident CAFs, those derived from bone marrow do not exhibit PDGFRα expression (Raz et al., 2018). One of the most abundant stromal cells in the TME, the CAF stromal compartment is often perpetuated by secreted factors from CAFs themselves, such as additional TGF-β, interleukin 6 (IL-6), platelet derived growth factor (PDGF), and chemokines like CXCL12 (Hill et al., 2020). Other tumor-secreted signaling modalities, such as non-canonical Wnt signaling via Wnt7a, can also reinforce TGF-β receptor signaling to sustain the CAF state (Avgustinova et al., 2016). ECM remodeling occurs through CAF deposition of ECM components such as collagen, fibronectin, and laminin, as well as via secretion of degrading matrix metalloproteases (MMPs). Busch *et al.* used single-cell methods to characterize the heterogeneity of fibroblasts and found that CAFs ‘primed’ by breast cancer cell lines had increased expression of MMP2, FSP1, Type I collagen, and fibronectin genes (Busch et al., 2017). The process of ECM degradation and deposition enables CAFs to alter tissue rigidity and form channels for cancer cell invasion, as observed in prostate cancer and squamous cell carcinoma through the involvement of integrins or RhoGTPases respectively (Gaggioli et al., 2007; Erdogan et al., 2017). It is plausible to presume that comparable mechanisms could be at play in breast cancer as well (Clark and Vignjevic, 2015; Mei et al., 2021).

Mounting evidence points towards a dynamic evolution of interactions between CAFs and tumor cells throughout the course of breast cancer progression. For instance, a specific subset of CAFs was found to have the unique ability to convert poorly metastatic MCF-7 cells into a metastatic state, implicating an SDF-1/CXCR4 axis as a crucial facilitator of this transformation (Sharma et al., 2021). Other studies demonstrate transglutaminase 2 (Tsg2)-rich microvesicles in facilitating cancer cell-CAF interactions for dissemination (Schwager et al., 2022). The presence of fibroblasts marked by low PDGFRα and high PDGFRβ associated with a higher risk of local recurrence in another study, where Notch signaling, linked to the disruption of the basement membrane, drives these fibroblasts to adopt a phenotype associated with increased expression of matrix-remodeling enzymes and TGF-β ligands (Strell et al., 2019). Interestingly, CAFs can also actively participate in conditioning the pre-metastatic and metastatic niche, facilitated by their movement from the primary tumor and subsequent mobilization to metastatic sites (Duda et al., 2010). Examining blood samples from both mouse models and cancer patients has uncovered the existence of circulating CAFs, found alongside CTCs, in solitary form, or in grouped clusters (Ao et al., 2015). Moreover, CAF subsets notably accumulate in axillary lymph nodes and enhance breast cancer cell invasion through distinct but complementary mechanisms (Pelon et al., 2020). In a panel of five TNBC patient tumors, another layer of CAF diversity within the stroma was revealed through scRNA sequencing, particularly within CAFs and novel perivascular-like cells displaying morphological, spatial, and functional properties, with such diversity contributing to immune evasion in the TME (Wu et al., 2020). The diverse attributes of CAFs are now becoming apparent and revealing the presence of distinct subcategories within them. A better comprehension of this stromal diversity will likely shed light on how CAFs add to the ever-changing intricacy and adaptive functionality of the tumor environment.

### Immune cells

Immunological alterations instigated by the tumor influence the development of metastatic disease in breast cancer, even prior to the point when cancer cells have disseminated to a secondary organ. There is growing evidence to suggest that the dynamic interplay among cancer cells, the tumor stroma, and the tumor immune ecosystem not only progresses with the disease but also significantly impacts the effectiveness of therapies. Certain tumors manage to circumvent the immune system’s control, thereby promoting tumor growth and metastasis. Conversely, others fall prey to an immune offensive, fine-tuned via the production of immunomodulatory ligands. The complexity and variability of the immune components within a tumor, coupled with their constant evolution during tumor progression, remain subjects of continuing investigation in the field of cancer research. Moreover, the way in which the composition of the tumor immune microenvironment is influenced by factors like cytokines and chemokines produced by the tumor, tumor oncogenes, and mutation landscapes is an ongoing investigation. Tumor necrosis factor α (TNFα)-activated MSCs expressed chemokines CXCL1, CXCL2, and CXCL5, recruiting chemokine receptor positive (CXCR2+) neutrophils to the TME. The interaction of these neutrophils and breast tumor cells results in increased expression of TGF-β, IL-6, MMP12, and MMP13 with increased metastasis to the lungs (Yu et al., 2017). Szczerba *et al.* leveraged the resolution of single cell RNA-sequencing to investigate the relationship between CTCs and white blood cells (WBCs) in breast cancer, where they identified many WBCs as Ly-6G+ pro-tumoral N2 neutrophils. Patients with these CTC-associated neutrophils had a significant decrease in progression-free survival. These clusters showed increased expression of vascular endothelial growth factor (VEGF) and proliferation signatures, suggesting an increased capacity for circulation entry, survival, and metastatic seeding (Szczerba et al., 2019). Neutrophils can also aid the spread of metastasis to the lungs by increasing the population of metastasis-initiating cancer cells through the release of leukotrienes (Wculek and Malanchi, 2015). Additionally, in the context of a lobular breast cancer model, it is been observed that neutrophil expansion via GCS-F and IL17-producing γδ T cells advance metastasis by suppressing a CD8^+^ T-cell immune response (Coffelt et al., 2015). Of course, tumor associated neutrophils (TANs) have been credited with both pro-tumor and anti-tumor functions (Granot et al., 2011; Finisguerra et al., 2015; Hedrick and Malanchi, 2022). Though certainly nuanced, their overall contribution to metastasis remains poorly understood.

Another major immune cell type associated with the TME are tumor associated macrophages (TAMs). There is significant evidence endorsing the role of classical inflammatory monocytes and macrophages in promoting metastasis. A pivotal investigation utilizing the MMTV-PyMT mouse model for breast cancer revealed that the absence of colony stimulating factor 1 (CSF-1), a factor necessary for the growth of CSF-1-dependent cells like monocytes and macrophages, led to slower transition of mammary tumors into metastasis (Lin et al., 2001). Real-time imagining identified a transient interaction between TAMs and tumor cells, where vascular permeability and tumor cell intravasation occur simultaneously upon direct contact with the vasculature (Harney et al., 2015). Recent findings also imply that metastasis is not just encouraged by TAMs at the original tumor location but is substantially furthered by macrophages and their precursors situated in areas prone to metastasis. Monocyte CSF-1 secreted from tumor cells activates the release and differentiation of monocytes into M0 macrophages. These macrophages can then be polarized by T-helper cells into either antitumoral M1 macrophages or protumor M2 macrophages (Qiu et al., 2018). Chemokines released by tumor and/or stromal cells, like CCL2, can attract chemokine receptor positive (CCR2+) inflammatory monocytes and M2 macrophages, which can assist in extravasation through monocyte-derived VEGF (Qian et al., 2011). In fact, many protumor functions of TAMs include the secretion of factors that promote angiogenesis (Dirkx et al., 2006) and/or activate ECM adherence through CCL18-mediated integrin clustering important for tumor cell invasion (Chen et al., 2011). Denardo and colleagues demonstrated that IL-4, specifically expressed by CD4^+^ T lymphocytes, reinforces a pro-tumor behavior in TAMs which activates epidermal growth factor receptor signaling to fuel metastasis to the lungs (Denardo et al., 2009). In fact, tumor-infiltrating lymphocytes (TILs), including CD4^+^ T regulatory cells (Tregs), can create an immunosuppressive environment by promoting immune tolerance to tumor cells (Bohling and Allison, 2008) and by eliminating members of the innate immune system like natural killer (NK) cells (Olkhanud et al., 2009). Regarding NK cells in models of breast cancer, CTC clusters, often forming the basis of polyclonal metastases, demonstrate higher resistance to NK cell elimination compared to individual CTCs, an aspect attributable to their elevated expression of cell-cell adhesion and epithelial genes, thus reducing the expression of NK cell activating ligands (Lo et al., 2020). Thus, NK cells, through their immunoediting capabilities, can influence the clonal evolution of metastasis by favoring polyclonal seeding over monoclonal metastases. Given the complex and evolving interplay among immune components within breast cancer, a continued pursuit of mechanisms governing these immune interactions and their modulation could unlock novel, more efficacious therapeutic approaches.

### Cancer associated adipocytes

Despite their widespread presence in the body, the contributions of adipocytes within the TME are often overlooked. Adipocytes, widely present within the breast TME, frequently encounter cancer cells; however, these cells are often perceived as mere passive entities without significant impact. Obesity, a known factor adversely impacting all intrinsic breast cancer subtypes, has multifaceted implications in disease progression, involving systemic metabolic alterations such as insulin resistance and changes in adipokines, and localized tissue inflammation (Neuhouser et al., 2015; Feola et al., 2017). Cancer-associated adipocytes (CAAs), reprogrammed by crosstalk with tumor cells, can secrete MMPs and inflammatory cytokines, contributing to more invasive breast tumors (Dirat et al., 2010; Dirat et al., 2011). Adipokines released by CAAs such as leptin, resistin, adiponectin, and visfatin can upregulate pro-inflammatory pathways, increase insulin resistance, and recruit immune cells that incur more invasive behavior in cancer cells (Tilg and Moschen, 2006). He *et al.* found that CAA-derived IL-6 and leptin upregulate expression of procollagen-lysine, 2-oxoglutarate 5-dioxygenase 2 (*PLOD2*), a lysyl hydroxylase necessary for collagen stability, and activate proliferation and migration pathways JAK/STAT3 and AKT (He et al., 2018). In fact, CAAs are known to remodel ECM through collagen deposition and contribute to the formation of stromal collagen networks at invasive fronts of breast tumors (Wei et al., 2019). Obesity can also create mechanical niches in mammary adipose tissue by augmenting the presence of myofibroblasts which leads to a denser, partially unfolded, and rigid ECM (Seo et al., 2015). Such changes not only perpetuate myofibroblast differentiation but can boost the malignant potential of mammary epithelial cells, offering new insights into the escalated risk and poor prognosis seen in obese breast cancer patients. Interestingly, the bone marrow microenvironment harbors bone marrow adipocytes (BMAs). In experimental models, breast cancer cells colonized human bone tissue, particularly the bone marrow adipose tissue compartment, in association with increasing levels of leptin and IL-1β, suggesting that bone marrow adipose tissue and its molecular signals are also potentially significant, yet often overlooked, components of the metastatic niche in breast cancer (Templeton et al., 2015). Considering the evidence that adipose tissue has the capacity to regulate metabolic activities both locally and distantly, it prompts intriguing exploration into the possible ways adipocytes could influence and mold different metastatic niches.

### Endothelial cells and pericytes

Solid tumors heavily rely on blood vessels for the provision of essential oxygen and nutrients, along with the removal of carbon dioxide and metabolic waste. Concurrently, these vessels act as key channels enabling malignant cells to spread to remote organs. The walls of blood vessels consist of two discrete types of cells, namely, endothelial cells and pericytes. Therefore, interactions between tumor cells and the endothelial cells and pericytes that comprise the vasculature play a key role in mediating disease progression (Sun et al., 2021). Tubes of endothelial cells recruited by VEGF create the vasculature basement membrane, supported by scaffolds of pericytes recruited by PDGF-β that interact with vasculature smooth muscle cells (vSMCs) (Gerhardt and Betsholtz, 2003; Lamalice et al., 2007). Secreted factors from breast cancer cells, such as VEGF, can activate neovascularization and alleviate hypoxia as tumors expand, providing key nutrients to support tumor outgrowth (Brown et al., 1999). IL6 secreted by tumor cells can activate STAT3 signaling, inducing HIF-1⍺ and VEGF and conditioning lymphatic endothelial cells to express CCL5 (Lee et al., 2014). Activation of STAT3 in endothelial cells has also been linked to an increase in cell adhesion molecules and tumor cell invasion (Kim et al., 2017). Interestingly, pericytes in the primary tumor that express endosialin enable metastasis to distant sites by aiding the intravasation of tumor cells through a process that is dependent on cell contact (Viski et al., 2016). This results in a surge in circulating tumor cells. Reflecting these preclinical tests, in separate groups of primary human breast cancers, a significant correlation is observed between heightened endosialin expression, augmented metastasis, and diminished patient survival (Viski et al., 2016). While impeding angiogenesis can decelerate tumor growth, it paradoxically might escalate metastasis (Ebos et al., 2009; Paez-Ribes et al., 2009). This contradiction could potentially be reconciled through vessel normalization in breast cancer, a process entailing augmented pericyte coverage, enhanced tumor vessel perfusion, diminished vascular permeability, and resultant alleviation of hypoxia. Interestingly, the interaction between CD4^+^ T cells and endothelial cells enhances pericyte coverage while reducing hypoxia, with corresponding data hinting at an impact on cancer prognosis in humans (Tian et al., 2017). Therapies bolstering the function of CD4^+^ T cells, like immune checkpoint blockade, could also induce a positive effect through normalization of the tumor vasculature.

## The metastatic niche

### The pre-metastatic niche

Tumor intrinsic and extrinsic factors play key roles in breast cancer metastasis by promoting invasion from the primary site and establishing supportive ecosystems at distant secondary sites. However, researchers now appreciate that the conditioning of these ecosystems can occur even before tumor cells leave the primary site. The concept of the pre-metastatic niche was first characterized in 2005 by Kaplan *et al.* with the discovery that bone marrow-derived VEGFR1 positive (VEGFR1+) hematopoietic progenitor cells accumulate in specific sites in the lungs prone to metastatic lesions (Kaplan et al., 2005). They found that these VEGFR1+ cells expressed VLA-4 (integrin α4β1) that binds fibronectin enriched in the lungs. The binding of VLA-4 to fibronectin then increased the expression of MMP9, breaking down the ECM and permeabilizing the lung vasculature. These clusters of fibronectin-anchored VEGFR1+ cells secreted CXCL12 and other growth factors, recruiting TME components like fibroblasts and later attracting CXCR4+ tumor cells (Kaplan et al., 2005).

### Extracellular vesicles in the premetastatic niche

The pre-metastatic niche represents the conditioned microenvironment prior to seeding of tumor cells, and extracellular vesicles (EVs) or extracellular particles (EVPs) derived from either plasma membrane budding (microvesicles) or through endocytic pathway release (exosomes and exomeres) carry important cargo to these distant sites (Lucotti et al., 2022). EVs and EVPs fuse with distant organ-specific cells to initiate an appropriately conditioned microenvironment conducive for tumor cell seeding. EV- or EVP-mediated conditioning results in several outcomes at distant sites, involving stromal cell recruitment/homing, ECM remodeling, growth factor secretion/release, and immunosuppression (Peinado et al., 2017). EVs enable long distance signaling between tumor cells at the primary site with bone marrow derived cells, stromal cells, and organ-specific cells at distant sites (Wang et al., 2021) The specific content of EVs contribute to the distinct patterns of organ tropism exhibited by tumor cells and certain cancer types. For instance, Hoshino *et al.* found that tumor-derived exosomes from both breast and prostate cancers contain distinct integrins that adhere to organ-specific cells and ECM components, such as Kupffer cells in the liver and fibroblasts and epithelial cells in the lungs (Hoshino et al., 2015). In translational studies by Borges, Jordan and colleagues, EVs from young women with breast cancer compared to healthy donors conferred invasive outcomes on tumor cells through focal adhesion kinase (FAK) signaling, demonstrating the use of proteomics to identify novel candidates for conditioning metastatic potential in this patient population (Jordan et al., 2020). In experimental models leveraging fluorescently labeled exosomes secreted by murine breast cancer lines, Wen and colleagues discovered that syngeneic intravenous injection of those exosomes into cancer naïve mice led to the accumulation of immunosuppressive MDSCs in both the lung and liver (Wen et al., 2016). In the brain, exosomal miRNA-503 release from breast cancer cells with loss of X-inactive specific transcript (XIST) was able to cross the blood-brain barrier and stimulate microglia to take on an immunosuppressive role, promoting brain metastases (Xing et al., 2018). Of course, the influence of EVs and EVPs on niche conditioning is dependent on their ability to reach distant sites of metastasis after they are shed from cells.

### Secreted factors in the premetastatic niche

Vasculature permeability, growth factor availability, hypoxia, angiogenesis, and metabolic reprogramming are all processes essential to premetastatic niche formation. Huang *et al.* demonstrated that angiopoietin 2 (Angpt2), MMP3, and MMP10 expressed by melanoma cells increase vascular instability in the lungs, and knock down of these genes significantly decreases the ability of MDA-MB-231 breast cancer cells to metastasize (Huang et al., 2009) (Figure 2C). In the bone niche, tumor cells instigate osteoclastic bone resorption, leading to TGF-β release from the bone matrix, which binds to the tumor cells and stimulates the expression of osteolytic factors like Parathyroid Hormone-Related Protein (PTHrP) (Yin et al., 1999) and Jagged1 (Sethi et al., 2011). Jagged1 facilitates osteoclastogenesis by engaging monocytes, and PTHrP induces Receptor Activator of Nuclear Factor kappa-B Ligand (RANKL) production in osteoblasts. RANKL, critical for osteoclast differentiation from monocytes and maturation from hematopoietic precursors, induces essential NF-κB signaling upon binding to RANK. Activated osteoclasts degrade the bone matrix, triggering a release of growth factors like TGF-β and perpetuating the cycle. TGF-β-induced Jagged1 amplifies this cycle by encouraging osteoblasts to produce tumor growth-promoting IL-6 (Esposito et al., 2018) (Figure 2B). This not only drives EMT and proliferation of tumor cells in the premetastatic niche, but also stimulates osteoblasts to generate new bone, in a vicious cycle of bone turnover. In breast cancer, Jagged1 expression can also drive this cycle through activation of the Notch signaling pathway and the release of IL-6 and TGF-β (Sethi et al., 2011). In the liver, high levels of tissue inhibitor of metalloproteinases 1 (TIMP-1) secreted by hepatocytes upregulate stromal cell-derived factor 1 (SDF-1), fibronectin, TGF-β, urokinase-type plasminogen activator (uPA), and S100A, inducing homing of breast cancer cells to this distant microenvironment (Seubert et al., 2015) (Figure 2D).

HIF secreted by osteoprogenitor cells in hypoxic niches in the bone can stimulate the CXCL12-CXCR4 axis that increases breast cancer proliferation and migration (Devignes et al., 2018). In the lungs, CAFs expressing long non-coding RNA, lncSNHG5, stabilize lncSNHG5-ZNF281-mediated CCL2/CCL5 secretion. This lncSNHG5-ZNF281-CCL2/CCL5 signaling axis prompted ZO-1 and cocculin expression and the stimulation of p38-MAPK signaling in endothelial cells, resulting in both vascular permeability and angiogenesis in pulmonary capillaries of the lung (Zeng et al., 2022). Besides angiogenesis, breast cancer secreted EVs containing microRNA miR-122 can increase nutrient availability by reprograming endothelial cells in the lung and brain to take up less glucose in the premetastatic niche (Fong et al., 2015) (Figure 2E). In fact, metabolite availability can play a significant role in ECM remodeling in the premetastatic niche. For example, the high availability of pyruvate in the lungs increases α-ketoglutarate production, increasing prolyl-4-hydroxylase (P4HA) enzymatic activity and the hydroxylation of collagen, increasing fibril strength necessary for receptor binding and crosslinking (Elia et al., 2019).

Together, these processes can adapt and change the microenvironment of secondary sites into the ideal “soil” to receive tumor cell “seeds,” even before their arrival. Once tumor cells disseminate from their primary site of origin and arrive within these idealized ecosystems, the establishment of the metastatic niche is initiated. Given the significant morbidity and mortality associated with metastasis, this interactive microenvironment for disseminated tumors cells holds extremely important therapeutic potential in cancer.

### The vascular niche

Metastasis is spatiotemporally guided by a plethora of signaling exchanges engaged by tumor cells and their microenvironment. Though metastasis encompasses a complex sequence of distinct events, intravasation and extravasation have common denominators. Endothelial cells typically serve as a barrier for the entry and exit of metastasizing disseminated tumor cells (DTCs) and are often compromised by the tumor-mediated expression of specific cell adhesion receptors and secreted factors which alter vessel permeability (Shenoy and Lu, 2016; Dhami et al., 2022). Most experimental metastasis approaches to assess metastatic progression of tumor cells focus on later events of the metastatic process; however, understanding early steps of organ colonization holds great promise from a therapeutic standpoint. Novel models employing microfluidics and intravital imaging in zebrafish embryos have served to interrogate the earliest steps of secondary site seeding by tumors cells, revealing essential insights into the molecular and mechanosignaling cues involved in successful extravasation (Follain et al., 2018). Despite this, our knowledge of the mechanisms contributing to metastatic colonization of common organs such as the brain, lungs, bone, and liver remain incompletely understood.

As discussed above, primary breast cancers shed CTCs into the peripheral blood circulation, and these cellular entities represent the precursors to metastatic disease (Krol et al., 2021). CTCs exhibit increased adhesion potential fostering intravascular arrest and facilitating extravasation to enable colonization at a secondary site. Osmani *et al.* revealed the extravasation stage consists of an initial weak arrest of CTCs followed by a more-stabilized adhesion between CTCs and endothelial cells that prevents detachment provoked by bloodstream shear forces (Osmani et al., 2019). Reports identified that early and weak intravascular arrest of highly metastatic CTCs is mediated by accumulated hyaluronic acid shed by tumor cells to support adhesion via the glycoprotein CD44 and integrin αvβ3, followed by a switch in adhesion receptor repertoire to integrin *α*5b1 for stable and more robust adhesion to the endothelial layer of vessels (Offeddu et al., 2021). The vascular architecture, composed primarily of an inner lining of endothelial cells in direct contact with pericytes surrounding blood capillaries (Bergers and Song, 2005), can be remodeled by secreted growth factors, cytokines, and loss of cell-junction integrity. Pericyte coverage is dynamic during tumor progression, and following adhesion, tumor cells weaken this barrier and actively remodel the endothelial layer by enhancing the permeability of the vasculature. Metastatic DTCs employ L1 cell adhesion molecule (L1-CAM) to mediate spread in a pericyte-like manner. L1-CAM expression in metastatic initiating cells activates YAP signaling by increasing β1 integrin-linked kinase (ILK) signaling, promoting cancer cells to dislodge pericytes and outgrow in bone marrow, brain, and lungs (Er et al., 2018).

Interestingly, DTCs occupy distinct sub-niches of the perivascular niche at the metastatic site, which are determinants of outgrowth potential. The lung and bone marrow sprouting microvasculature sustains breast cancer quiescence via endothelium-derived secretion of thrombospondin-1 (TSP-1). In contrast, the sprouting neovasculature promotes DTC growth by enhancing TGF-β1 signaling (Ghajar et al., 2013a). Recent findings have revealed unique insights into the cellular and molecular mechanisms driving DTCs quiescence during brain metastasis. In the brain, pericytes and endothelial cells line the vasculature, and astrocytes conform to the perivascular niche (Charles and Holland, 2010). Studies further implicated specialized astrocytic structures called endfeet, expressing laminin-211, in suppression of DTC outgrowth. Astrocytic laminin-211 deposition in the basement membrane stimulated a DTC quiescent state, triggering dystroglycan signaling and YAP membrane sequestration to control this state of dormancy (Dai et al., 2021). Furthermore, ECM proteomics has revealed that dormant cancer cells also assemble a Type III collagen-enriched non-linear ECM niche (Di Martino et al., 2021).

Transcriptomic and proteomic analyses have identified fibronectin and tenascin-C (TNC) as two key ECM constituents important for regulating the fitness of tumors cells in the perivascular niche (Hebert et al., 2020; Hongu et al., 2022). Analysis of breast cancer tissue samples revealed that TNC expression correlates with metastatic relapse in the lung. Mechanistically, TNC expression enhances expression of Leucine-rich repeat-containing G protein-coupled receptor 5 (Lgr5), potentiating Wnt/β-catenin signaling (Oskarsson et al., 2011). Concomitantly, TNC also augments Notch signaling by promoting the RNA-binding protein Musashi homolog 1 (MSI1) expression. TNC engagement with both the Wnt and Notch signaling pathways thus supports metastatic fitness of DTCs during early seeding stages in the lungs**.** Although several studies highlight the importance of the perivascular niche in metastasis, we are only just beginning to understand the nature of this local niche with respect to organ tropism, dormancy, and therapeutic resistance. Recent studies targeting integrin-mediated interactions between DTCs and the perivasculature to sensitize dormant cells to chemotherapy highlight the promising opportunity to target this niche in the clinical setting of metastasis (Carlson et al., 2019). How this vascular niche differs across organs, as well as illumination of mechanisms regulating tissue-specific interactions of DTCs with tissue-resident cells of the secondary organ, should yield important insights regarding distant tissue microenvironments and approaches to target organ-specific metastases.

## Tumor cell dormancy

Despite some triumphs associated with targeted therapies and immunotherapies, metastasis and drug resistance are common outcomes for patients. Metastatic recurrence typically happens long after primary tumor removal (Friedl and Herfarth, 1992; Leman and Mac Kie, 2003) and tumor relapses often arise from minimal residual cancer cells after systemic therapies (Aguirre-Ghiso, 2007; Demicheli et al., 2007). These observations indicate a long-term growth arrest program occurs in a subset of cancer cells, likely contributing to tumor metastases and drug resistance. Even in the case of minimal residual disease in both primary and distant sites following targeted therapy, such cells can persist in a dormant state despite sufficient vascularization and the lack of adaptive immunity (Abravanel et al., 2015; Ruth et al., 2021). They possess the capacity to re-engage with the cell cycle after prolonged periods of latency, leading to the development of recurrent tumors. Based on experimental models, a large portion of metastatic cancer cells fail to survive shear stresses within the circulatory or lymphatic systems after leaving the primary tumor, and only a very small number of DTCs (<0.02%) eventually develop macro-metastasis in secondary organs (Luzzi et al., 1998; Cameron et al., 2000; Wong et al., 2001). In these experiments, some tumor cells were found to enter a dormant state to survive the stress encountered within the circulation and upon arrival within distant organs. This dormant state typically occurs following 1) insufficient oxygen and nutrients, 2) anti-tumor immune surveillance, and 3) lack of supportive stroma. Though dormant tumor cells resemble a quiescent stem-like state accompanied by G0-G1 cell cycle arrest and are capable of reactivation to proliferate or differentiate (Pelayo et al., 2006), their biology is quite distinct from stem cells. Multiple signaling pathways are implicated in the regulation of cell dormancy, and this state is also intricately controlled by the local environment in which these cells reside (Phan and Croucher, 2020). DTCs will eventually exit dormancy, initiate proliferation, and eventually develop into macrometastases (Linde et al., 2016). However, elucidation of the precise mechanisms regulating emergence from this state is still in its infancy. The duration of the dormant state can vary from months to years, and this uncertain timing of reactivation remains a mystery today (Quesnel, 2013). In the following section, we introduce some of the cell intrinsic and extrinsic cues regulating tumor cell dormancy in metastasis.

### Signaling pathways implicated in dormancy maintenance and escape

#### Urokinase receptor (uPAR), extracellular signal-regulated kinase (ERK), and p38 mitogen-activated protein kinase (p38) signaling

**FIGURE 3:**
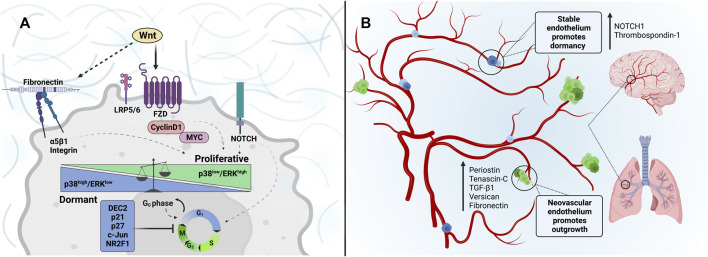
Dormancy and reawakening of cancer cells: Extracellular matrix cues associated with tumor cell entrance into and escape from dormancy within the metastatic niche. **(A)** Wnt-signaling through Frizzled and LRP5/6 receptors induces the expression of cyclin D1 and MYC, promoting G0-G1 transition and G1-S, S-G2, and G2-M cell cycles. Similarly, a fibronectin signaling axis activation through α5β1-integrins can be regulated by Wnt signaling to increase invasion and tumor outgrowth, while inhibition impedes stable adhesion and can sustain tumor dormancy. Such tumor microenvironment and ECM alterations can modulate mechanisms underlying dormancy state fate, such as p38/ERK signaling. The p38^high^/ERK^low^ ratio is associated with inducing G0-G1 growth arrest. While cancer cells with p38^low^/ERK^high^ are highly proliferative. **(B)** The perivascular niche is depicted. The tumor-promoting nature of the sprouting microvasculature of the lung and brain is characterized by enhanced expression of matrix-associated factors such as Periostin, Tenascin-C, TGF-β1, Versican, and Fibronectin. DTCs that reside in the sprouting neovasculature are prompted to proliferate in response to this conducive niche. In contrast, the stable vascular endothelium is rich in NOTCH1 and Thrombospondin-1, promoting tumor-cell dormancy.

One of the most widely studied mechanisms in tumor dormancy and proliferation is the ERK and p38 signaling pathways (Figure 3A). Strong ERK activation is observed in highly malignant tumors, and constitutive activation of ERK signaling is critical for G0-G1-S-phase cell cycle transition and cell proliferation (Chambard et al., 2007). While ERK signaling promotes growth, p38 signaling acts as an inhibitory system to prevent cell proliferation by inducing G0-G1 arrest and long-term cell dormancy in tumor cells (Bulavin and Fornace, 2004). The balance of ERK/p38 signaling in proliferation and dormancy has been observed in several types of cancer. One strong piece of evidence supporting this balance is that an ERK/p38 luciferase reporter demonstrated high ERK/p38 ratios in growing tumors but low ERK/p38 ratios in dormant/non-proliferative tumor cells (Aguirre-Ghiso et al., 2003). Genetic and pharmacological tools were utilized to alter the ERK/p38 ratio, and results confirmed that ERK/p38 signaling could dictate dormant and proliferative states (Sosa et al., 2011).

The ERK and p38 signaling pathway is largely modulated by uPA system and ECM (Lee et al., 2003). uPA is secreted by various types of cells in the TME and induces a proteolytic cascade that converts plasminogen into plasmin to degrade ECM components (D'Alessio and Blasi, 2009). Besides this proteolytic function, uPA and its receptor (uPAR) can lead to ligand-independent activation of EGFR and interact with integrin β1 and its ligand fibronectin to trigger downstream signaling which recruits FAK (Aguirre Ghiso et al., 1999; Aguirre Ghiso, 2002; Liu et al., 2002). Activated EGFR and integrin signaling can enhance ERK activity while reducing p38 activity, hence promoting proliferation and migration (Sawai et al., 2005; Barkan et al., 2010; Choi et al., 2018). Independent studies also demonstrate that disruption of integrin β1 or FAK triggers dormancy in mammary epithelial cells, breast cancer cells, or Hep3 cells (Weaver et al., 1997; Aguirre Ghiso et al., 1999). High ERK activation can also maintain uPAR levels through a positive feedback loop, which is commonly observed in proliferating and non-dormant tumor cells both *in vivo* and *in vitro* (Nguyen et al., 1999; Ma et al., 2001).

There is evidence that dormant tumor cells can harbor significantly lower levels of uPA and uPAR (Aguirre-Ghiso et al., 2001). Once uPA/uPAR is downregulated, fibronectin-integrin-EGFR signaling is diminished and the balance between ERK and p38 is switched to favor p38-induced cell dormancy. Previous reports demonstrate that p38 is essential for the phosphorylation of p53 and its tumor suppressive functions (Perfettini et al., 2005). Various transcription factors were discovered downstream of p38, including DEC2, p21, p27, c-Jun, and NR2F1, mediating G0-G1 arrest and cellular quiescence (Adam et al., 2009; Sosa et al., 2011; Bragado et al., 2013). Among these transcription factors, NR2F1 plays a key role in dormancy induction by enhancing the expression of a CDK inhibitor p27 through HIF1, SOX9, and RARβ (Sosa et al., 2015). NR2F1 also induces the expression of NANOG which is critical for cancer cell stemness and heterogeneity (Sosa et al., 2015). Finally, NR2F1 is essential for hypoxia-induced tumor cell dormancy (Fluegen et al., 2017). These molecular studies are consistent with a clinical result showing that NR2F1 expression positively correlates with relapse time and patient survival (Borgen et al., 2018). In addition to cell cycle regulation, p38 contributes to ER stress modulation through the unfolded protein response (UPR) sensor ATF6α. ATF6α enhances mTOR survival signals by increasing the expression of a small GTPase, Rheb (Ranganathan et al., 2006a; Schewe and Aguirre-Ghiso, 2008). Another study in tumor ER stress demonstrated that the p38-dependent PERK-eIF2α pathway facilitates cell cycle arrest and survival (Liang et al., 2006; Jiang et al., 2014). Moreover, p38 activation transcriptionally promotes the ER chaperone BiP which inhibits Bax to favor tumor drug resistance and cell survival (Ranganathan et al., 2006b). Taken together, a low ERK/p38 ratio switches tumor cells to a dormant state while turning on programs necessary to confer treatment resistance.

#### Transforming growth factor-β (TGF-β) and bone morphogenetic protein (BMP) signaling

Many studies implicate the TGF-β family as a key regulator of induction and maintenance of the dormancy state. TGF-β and BMP pathways are activated by the binding of ligands to Type I and Type II TGF-β receptors and the initiation of downstream SMAD-dependent or -independent pathways (Rahman et al., 2015). TGF-β and BMP signals are pleiotropic in nature, so they exhibit context-dependent roles in tumor dormancy based on the cell types, secondary organs, and microenvironment encountered (Prunier et al., 2019). Within the bone marrow, elevated TGF-β and BMP pathway activation can maintain dormancy in head and neck squamous cell and prostate cancers through growth arrest-specific 6 (GAS6) and its receptor Axl (Bragado et al., 2013; Yumoto et al., 2016). Similarly, BMP-7 binding to its receptor, BMPR2, is necessary for dormancy and metastasis of prostate cancer stem-like cells in the bone microenvironment (Kobayashi et al., 2011). The same is true for breast cancer. Periarteriolar BM-resident NG2+/Nestin+ MSCs induce a dormant state for disseminated breast tumor cells through TGF-β2 and BMP7 cellular program relayed by TGFBRII and BMPRII, respectively (Nobre et al., 2021). A secreted antagonist of TGF-β ligands, Coco, blocks paracrine BMP signaling and downstream GATA3 to enhance the self-renewal capability and reactivation of dormant breast cancer cells in the lungs (Gao et al., 2012). Additional studies also demonstrate that TGF-β signaling can increase p38 activity while blocking ERK activity to enable entry into a dormant state (Yu et al., 2002).

Interestingly, TGF-β1 is secreted within tip endothelial cells during neoangiogenesis but maintained at a low level around the mature blood vessels (Allinson et al., 2012). This localized TGF-β activation increases periostin expression, thereby triggering dormant breast cancer cells to enter a proliferative state (Ghajar et al., 2013b). Mature blood vessels, on the other hand, express low levels of periostin and high level of TSP-1, supporting the perivascular quiescence of breast cancer cells (Kazerounian et al., 2008; Ghajar et al., 2013b). TSP-1, TGF-β2, and p53 are also significantly reduced when the leukemia inhibitory factor receptor (LIFR) is silenced (Johnson et al., 2016). It is well known that LIFR and VEGF signals are triggered by hypoxia in a HIF-independent manner (Shweiki et al., 1992; Jeong et al., 2007; Johnson et al., 2016). Therefore, TGF-β signaling is a likely mediator of hypoxia-induced dormancy maintenance (Figure 3B).

### Wnt signaling

Wnt signaling is a multifaceted signaling pathway based on the combination of cell-surface receptors and intracellular signaling constituents engaged, dictating either β-catenin-dependent or β-catenin-independent activation states. Canonical Wnt signaling through Frizzled and LRP5/6 receptors stabilizes β-catenin to induce the expression of cell cycle regulators cyclin D1 and MYC (Mateyak et al., 1999; Sutherland and Musgrove, 2002; Alao, 2007; García-Gutiérrez et al., 2019). Although Cyclin D1 and MYC are not Wnt pathway-specific, such β-catenin inputs can converge on cell cycle and survival programs for the cell. In breast cancer, latency competent cancer cells actively impose a slow-cycling state by producing an extracellular Wnt modulator, DKK1, concomitantly linked to their ability to evade innate immunity within the lung niche (Malladi et al., 2016). In independent studies, another extracellular Wnt modulator, Srfp2, is triggered from breast cancer cells upon engagement with lung AT1 alveolar epithelial cells, reinforcing an integrin-fibronectin survival loop important for DTCs (Montagner et al., 2020). ECM composition plays an important role in metastatic dormancy and provides a critical reservoir for Wnt ligand availability within the metastatic niche. TNC, one such ECM protein, is secreted by disseminated TNBC tumor cells (MDA-231 LM2) in the lung niche, augmenting the activation of Wnt/β-catenin signaling to promote the reactivation dormant breast cancer cells (Oskarsson et al., 2011). Such experimental findings are supported by clinical data that shows a significant correlation between TNC expression levels and the risk of breast cancer recurrence in the lungs (Calvo et al., 2008). Periostin, a scaffold protein in the bone, can also activate Wnt/β-catenin signaling and contribute to dormancy exit (Ikeda-Iwabu et al., 2021) and play a very similar role in breast cancer dormancy emergence (Ghajar et al., 2013b). Noncanonical β-catenin-independent Wnt signaling can also inhibit Wnt/β-catenin signals and ECM cues to determine the dormancy state of tumor cells. Noncanonical Wnt5a supports prostate cancer dormancy in the bone microenvironment via its cognate receptor Ror2 and induction of downstream Siah E3 Ubiquitin Protein Ligase 2 (SIAH2) (Ren et al., 2019). In genetically engineered mouse models of TNBC, Wnt/Ror2 signaling also regulates tumor cell-intrinsic collagen production and fibronectin/integrin signaling to modulate tumor cell dissemiation (Si et al., 2022). Further exploration of such integrin-matrix interactions, mediated by Wnt, represents a potential therapeutic vulnerability in breast cancer metastasis by leveraging such findings within the emergent dormancy niche.

### Notch signaling

Notch signaling, mediated by four receptors (Notch 1–4), is widely involved in juxtacrine interactions across development, stem cell self-renewal programs, proliferation and differentiation cues, and other cellular processes (Kopan, 2012). Analyses in breast cancer patients revealed a positive correlation between breast cancer recurrence and the expression levels of Notch1 and its ligand Jagged1 (Yuan et al., 2015; Zhong et al., 2016). In breast cancer, the HER2+ subtype harbors low Notch1 activity and experimental modeling in mouse and cell lines demonstrated the ability of HER2 to negatively regulate Notch1 activity (Osipo et al., 2008). This finding suggests that Notch1 signaling is activated in response to HER2 blockade and triggers anti-HER2 drug resistance. Indeed, genetic or pharmacologic inhibition of Notch1 enhanced trastuzumab (an anti-HER2 therapy) sensitivity and diminished recurrence from residual dormant breast cancer cells after trastuzumab treatment (Pandya et al., 2011). A possible mechanism regulating the crosstalk between HER2 and Notch1 is through ERK pathway activation. HER2+ breast cancer cells can activate ERK signaling which decreases Notch1 cleavage, thus stabilizing survivin to promote HER2+ cell survival (Ju et al., 2013). Besides HER2, Notch1 is also modulated by EGFR. EGFR can diminish Notch1 signaling via MAPK/MEK/ERK activation in squamous-cell carcinoma (Kolev et al., 2008). On the other hand, Notch and EGFR signaling pathways are both elevated in the context of TNBC and a compensatory Notch-AKT signaling loop is proposed to cause resistance to EGFR inhibition therapies (Bianco et al., 2003; Sergina et al., 2007; Meurette et al., 2009; Dong et al., 2010; Ercan et al., 2012). Notch2 can also contribute to cancer dormancy and metastasis. Notch2 maintained breast cancer dormancy in the bone microenvironment, by means of hematopoietic stem cell mimicry, and γ-secretase inhibition of Notch activity prompted dormancy exit and metastatic colonization (Capulli et al., 2019).

### Immune and inflammatory signaling

Cancer immune surveillance represents a central host protection process to impede the initiation and spread of tumor cells and preserve cellular homeostasis. Immunosurveillance within secondary organs is the basis for durable cancer cell dormancy (Wang et al., 2019); however, various biological factors in metastasis result in the waning of this equilibrium over time to prompt dormancy exit and metastatic relapse (Kim et al., 2007). Immune-suppressive cells combined with reciprocal cell-cell signals facilitate the immune escape of cancer cells, enabling their metastatic emergence in distant organs (Goddard et al., 2018). Multiple immune cells are involved in this inflammation-mediated dormancy maintenance and escape in breast cancer. CD4^+^ and CD8^+^ T cells were reported to promote dormancy by both immune dependent and independent functions. In breast cancer patients, dormant tumor cells in the bone marrow are closely associated with increased presence of CD4^+^ and CD8^+^ T cells (Feuerer et al., 2001). Moreover, extended disease-free survival times correlated with a CD39^+^PD1^+^CD8^+^ T cell population rather than total CD^+^ T cells. In experimental models, production of TNFα and INFγ cytokines by this T-cell population maintains this state of dormancy (Tallon de Lara et al., 2021).

The cumulative exchange between the tumor and immune microenvironment has revealed that the immune landscape in a tumor evolves from inhibitory to permissive, often accompanied by a change in repertoire of immune cell effectors. In addition to effector T cells, neutrophils are responsible for the degradation of TSP-1 and dormancy escape in chronic inflammation induced by lipopolysaccharide (LPS) (El Rayes et al., 2015). Following LPS-induced inflammation in the lung, a ZEB1-mediated EMT program can contribute to the exit from tumor cell dormancy (De Cock et al., 2016). Contributing to this chronic inflammation, neutrophil recruitment to the lung niche prompts the formation of neutrophil extracellular traps (NETs) to awaken dormant breast tumor cells by locally sourcing proteases to remodel laminin-111 ECM (Albrengues et al., 2018). ECM composition can certainly shift the balance between dormant and proliferative states for disseminated tumor cells within the lung (Barkan et al., 2010). In other cancers, neutrophils also remodel fibronectin in the ECM and trigger integrin α5β1 to increase the ERK/p38 ratio (Monti et al., 2017), which awakens dormant cancer cells. NK cell presence is also expanded in dormant *versus* progressing metastatic niches (Wu et al., 2013; Brodbeck et al., 2014; Correia et al., 2021). In breast cancer, dormant DTC reservoirs in the liver are controlled by INFγ+ NK cells (Correia et al., 2021), while inflammation provoked by liver injury allows the accumulation of hepatic stellate cells (aHSCs) to normalize NK cell presence and enable dormancy exit (Correia et al., 2021).

Inflammatory signals, especially chronic inflammation, may play a critical role in deciding the dormant state of cancer cells. Inflammation is correlated with breast cancer recurrence in patients, and this inflammation link to recurrence is present within other cancer types (Cole, 2009; Kinoshita and Goto, 2021; Choi and Hwang, 2022). Inflammatory cytokines (e.g., IL-6) show promising results as potential prognostic markers to predict the risk of cancer recurrence and patient death (Duffy et al., 2008; Kita et al., 2011). IL-6/JAK1/STAT3 signaling has been recognized as a key mechanism for cancer drug resistance and cancer stemness (Ara et al., 2013; Manore et al., 2022). Consistently, IL-6, LIF, and STAT3 were found to regulate breast cancer dormancy in the bone (Johnson et al., 2016). Another inflammatory cytokine, IL-11, was demonstrated to modulate the RANK/RANKL pathway, thereby influencing bone resorption to modulate cancer dormancy in bones (Giuliani et al., 2004; McCoy et al., 2013). Additionally, inflammatory chemokine signaling is involved in breast cancer dormancy escape. CXCR4 is downregulated in dormant breast cancer cells in the lungs, and the upregulation of CXCR4 with its ligand, CXCL12, promotes AKT signaling and proliferation (Zhao et al., 2008; Nobutani et al., 2015).

## Targeting the metastatic niche

Though systemic and targeted therapies are efficacious in most non-advanced breast cancers, metastatic disease eventually reappears months to years after successful treatment of the primary tumor. A diagnosis of metastatic disease is often terminal for these patients, and these prognostic odds must change. The processes that determine the timing and site of relapse remain a puzzle in cancer research. Given that breast cancer remains the most diagnosed cancer among women worldwide, identifying tumor-intrinsic and extrinsic drivers and predictors of recurrence and metastasis is pivotal to challenging the paradigm in patient care. Axillary lymph node status remains the most important predictor of breast cancer patient outcomes, yet up to 30% of node-negative cases metastasize (Zemni et al., 2017). Recent advancements propose machine learning systems, such as artificial intelligence, to provide valuable insights into the early detection of metastasis using blood profiling and mammogram data (Rafique et al., 2021). In the clinic, micrometastases are classified based on a size between 0.2–2 mm in diameter. It presents considerable challenges to detect these micrometastases using standard ultrasound and MRI-based imaging, which lack appropriate sensitivity to detect clinically indolent lesions. Approaches focused on increasing the specificity of detecting micrometastatic lesions have employed mannose and near-infrared dye IR780 conjugates (MR780) to detect M2 TAMs shown to be found in metastatic lymph nodes as an alternate metastasis detection approach (Zhao et al., 2022).

In instances of hematogenous dissemination, liquid biopsies serve as a noninvasive tool to monitor disease burden. Recent efforts have focused on the development of digital polymerase chain reaction (PCR) for quantitative assessment of mutation status identified in circulating tumor DNA (ctDNA) to support current clinical diagnostics and detection of therapeutic relapse *in lieu* of repeat tumor biopsy (Turner et al., 2020; Fackler et al., 2022). Predicting if a patient will relapse is an immense challenge in the clinic that still needs additional research. Moreover, targeting micrometastasis poses an even greater hurdle due to their sparsity and unknown distribution in distant organs, as well as insufficient knowledge of their genetic and phenotypic traits prior to progression to overt metastatic disease. While targeting different stages of metastatic progression is challenging, recent efforts have taken advantage of antibody-based therapies and optimized them to increase selectivity and penetrance in organs of high metastatic incidence. Antibody-drug conjugates (ADCs) represent a promising approach exemplified by the DESTINY-Breast03 trial of trastuzumab deruxtecan, which demonstrated substantial improvements in overall patient survival for pre-treated metastatic HER2-+ breast cancer (Hurvitz et al., 2023). Experimentally, antibody-drug conjugate Trastuzumab with a hydroxyapatite-binding bone-homing peptide exhibits enhanced therapeutic efficacy *in vivo*, selectively delivering the agent to the bone metastatic niche (Tian et al., 2022). Overall, many promising new strategies are in development to improve the treatment of metastatic breast cancer which rely on features specific to the metastatic niche to improve therapeutic delivery and increase sensitivity.

## Closing the gap: charting the unmapped territories of breast cancer metastasis

The seemingly unpredictable nature of metastatic relapse in breast and other cancers exemplifies both a challenge and opportunity clinically. Understanding the biology of the metastatic niche, from dormancy to emergence, is essential to appropriately validate the utility of pre-clinical models that accurately reflect the clinical course of metastatic progression for patients and achieve therapeutic success. This includes immune competent models that allow inclusion of tumor extrinsic cell types and consider how inflammation and the immune landscape participate in context. Such models will be imperative to identify therapeutic opportunities based on the biology of metastasis gleaned from rigorous experimental inquiry. The application of single cell and spatial transcriptomics and proteomics technologies will undoubtedly provide immense value in both experimental and clinical settings of metastasis to help deconvolute the cellular landscape encompassing metastatic disease. These spatial tissue atlases are just beginning to be constructed and utilized effectively. Access to these technologies holds great promise in navigating emergent data from clinical trials and patient-centered studies to determine how cellular composition and transcriptomic hallmarks contribute to encompassing metastasis biology. The process of breast cancer metastasis, which is still fraught with many unknowns, heavily relies on complex mechanisms. Among these, the role of disseminated tumor cells—their origins and how they contribute to metastasis—stands as a key area requiring deeper understanding. The mysterious processes governing cancer cell dormancy and subsequent reactivation also present intriguing research prospects. Noteworthy is the complex interplay between cancer cells and their microenvironment, a relationship that may potentially shed light on tumor progression, metastasis, and resistance to therapy. This is particularly relevant given that breast cancer’s preference to metastasize to certain organs—a phenomenon known as organotropism—has yet to be completely understood. The challenges in metastatic breast cancer treatment, particularly therapy resistance, underscore the urgent need for comprehensive inquiry. Understanding both intrinsic and acquired resistance to cancer therapies, stemming from complex cellular and molecular mechanisms, remains a significant challenge in oncology. This complexity is further compounded by the substantial heterogeneity of breast cancers, with variations seen both between different patients and within an individual patient’s tumor. As the role of immunotherapy in cancer treatment expands, deciphering why certain tumors either do not respond or develop resistance to these therapies is emerging as a critical area of investigation. Identifying accurate predictive and prognostic biomarkers could pave the way for earlier and more precise interventions, in addition to potentially preventing metastasis. There is a growing momentum towards personalized therapies tailored to individual genetic profiles of patients and their tumors. This could revolutionize treatment outcomes, setting a new paradigm in the fight against metastatic breast cancer. However, these areas of research are still under development and require further investigation for their full potential to be realized. Success in the field of breast cancer metastasis will most likely occur from thoughtful discourse between researchers, clinicians, and patient advocates to generate new hypotheses and clinical trial designs directed at targeting the molecular underpinnings of metastasis to improve patient survival.
